# Rapid accommodations for university students with concussion are feasible: nationwide return-to-learn perspectives of accessibility staff

**DOI:** 10.3389/fneur.2026.1770164

**Published:** 2026-08-05

**Authors:** Zachary W. Bevilacqua, Nicole Mucica, Kaitlyn Bjelko, Christina Finn

**Affiliations:** 1 Department of Kinesiology, Sport Studies and Physical Education, SUNY Brockport, Brockport, NY, United States; 2 Academic Success Center, SUNY Brockport, Brockport, NY, United States; 3 Department of Occupational Therapy, New York Institute of Technology, New York, NY, United States

**Keywords:** accessibility, college, concussion, disability, return to learn, RTL, RTLU

## Abstract

**Introduction:**

Current recommendations for concussed college students promote rapid implementation of accommodations (<3 days), however, it is unknown whether college accessibility staff find this recommendation feasible. Thus, this research aimed to gather the perceptions of accessibility staff regarding implementation of accommodations for students who have sustained concussions.

**Methods:**

A cross-sectional design was used to distribute a nationwide survey to accessibility staff working on college campuses (*n* = 87). Distribution methods included a nationwide listserv along with hand searching institutional websites. Survey recruitment took place electronically across the United States between 1/2025–7/2025.

**Results:**

Total sample mean and median feasibility scores were 7.88 and 8, respectively (0–10 scale). Seventy percent of participants reported the ability to accommodate a concussion in <3 days, with 89% accomplishing this task when provided 4 days. Limited student reporting (28%) and streamlined communication with medical providers (15%) were most frequently noted as variables impeding the feasibility of this task. No significant associations were found between feasibility scores and location (*p* = 0.840) or institution size (*p* = 0.094). No significant associations were found between perceived barriers and location (*p* = 0.091) or size of institution (*p* = 0.635).

**Discussion:**

Most sampled accessibility staff across the Unted States find rapid (<3d) accommodations to be a feasible task, supporting our understanding and confidence in recent RTLU guidance. These data reveal an existing capacity to perform RTLU recommendations, encouraging feasibility.

## 1 Introduction

Concussion is defined as a mild traumatic brain injury caused by external forces, causing physiological disruption of brain function resulting in the onset of neurologic signs, symptoms, and functional limitations (1). Sequelae may include headache, dizziness, visual problems, and difficulty concentrating (1, 2). As a result, individuals may have difficulty returning to school and may report greater perceived academic problems following a concussion (3). Recent evidence suggests that gradual return to learn, interdisciplinary approaches, and implementation of accommodations are best practices to facilitate returning to grade school and high school following a concussion (2, 4, 5). Thus, published guidelines provide recommendations for timelines, accommodations and guidance to support return to learn at the grade- and high-school levels (2, 6).

Recently, authors have cited numerous pragmatic and environmental differences as a foundation for why existing return to learn protocols should not extend from primary and secondary schools into higher education (7, 8). For instance, college inherently fosters a competitive environment, which compounded with the autonomous nature of higher education can lead to increased stress and anxiety (9, 10), acculturative stressors (11, 12), and feelings of not belonging (13). Success in higher education also demands considerable maturation, as students lack the oversight otherwise seen in primary and secondary schools. Under more trying conditions, such as concussion, students will face other unique challenges. For example, university academics are faster paced than middle-, high school, and the like, meaning class absences and accumulating work due to concussion will quickly invoke anxious responses (14). Furthermore, primary and secondary school teachers are encouraged to implement academic adjustments for concussion ad libitum (6, 15, 16), whereas university faculty have identified themselves as unfit to perform this responsibility (7). Given the unique nature of the collegiate experience and approach to education, the return to learn path for college and university students should be distinct from K-12 recommendations.

Academic support for university students ultimately requires faculty involvement and follow-through, thus, evidence was gathered to understand faculty perspectives of return to learn. Themes including “awareness of concussion”, “legitimacy/proof of the injury” and “willingness to accommodate” were found to guide faculty decision-making when asked to accommodate a concussed student (7). Faculty from other institutions and disciplines brought about themes of “legitimacy/proof of injury” and “empathy/willing to help,” replicating findings from initial grounded theory work (17). Various other data provide further color to faculty behaviors. For instance, 62% of faculty (*n* = 220) reported how they would require a disability services note prior to accommodating a student (18). Others supported accommodations 100% of the time when documentation from an accommodations office was available, versus 75% of the time when documentation was not (17). Registering with the campus disability office and having a documented disability were identified as important to faculty when asked to accommodate students, citing fairness amongst students as the reason (19), matching the rationale of other cohorts (7, 17). Student experiences convey a parallel story, reporting faculty skepticism towards students with similar “invisible” disabilities (20, 21), indicating that these faculty perspectives are not isolated to interactions with concussed students, but are well triangulated throughout various other contexts. Overall, faculty appear to require documentation before allowing accommodations, emphasizing the role of the campus accessibility/disability services office within return to learn. Faculty are required by law to allow reasonable accommodations when requested by campus accessibility for a student. However, the acute nature of concussion and pace of university academics means that accommodations will be needed quickly post-injury.

A successful return to learn path for college and university students (RTLU) is expected when accommodations are sought, implemented quickly (<3 days), and are facilitated by a collaboration between the healthcare provider and campus accessibility office (22). To operationalize these tenets, an RTLU protocol was introduced by Bevilacqua and McPherson, guiding students towards accommodations in <3 days following the diagnosis of a concussion (22). The abbreviated timeframe considers the fast-paced nature of college academics and accumulation of class content (23), along with consensus recommendations to re-introduce academics no later than 48 h post-injury (2). Furthermore, this protocol capitalizes on existing university infrastructure and legal precedents surrounding student accommodations and interprofessional collaboration in higher education to support the protocol at an implementation level. Data from athletic trainers agrees that an RTLU process should be initiated by the healthcare provider, suggesting that these professionals would find value in novel RTLU procedures (14, 24). However, it is unclear whether other end-users, such as accessibility services staff, find suggested procedures and timeframes to be feasible; thus, we solicited the perspectives of accessibility staff in higher education across the United States through a nationwide survey effort. Specifically, we sought to better understand the perceived feasibility of providing concussed students with accessibility sponsored accommodations in < 3 days, in addition to any variables that could impede or facilitate this task.

## 2 Materials and methods

### 2.1 Recruitment

A Qualtrics survey (Qualtrics Survey Software®) was distributed by email to Accessibility Staff across the United States between 1/2025–7/2025. Potential participants were identified by a nationwide listserv accessed through the New York State Disability Services Council^1^, in addition to hand-searching institutional websites with the intent of reaching participants from states that recorded a low response rate. Participants were allowed to volunteer for the study if they were currently working in a college/university Accessibility Office, or like entity, that provides 504 accommodations to students through the Americans with Disabilities Act. The full list of hand-searched institutions is found in Supplemental material 1. Participants received a $10 electronic Amazon gift card for participation. All study materials and procedures received approval from participating Institutional Review Boards.

### 2.2 Survey

Existing literature suggests that provision of formal accommodations and a systematic return to learn process implemented in a timely manner best supports return to learning at the university level following concussion (14, 17, 18). However, there are often barriers at the institutional level that can create challenges to implementation (7, 25). As a result, our survey was designed to explore existing practices, access to training and education, and barriers to implementation of accommodations following concussion. The survey was designed to assess four primary constructs:

*Current practices utilized by offices of accessibility* including implementation of accommodations, timeframe, and procedures.

*Perceived feasibility* of implementing accommodations and return to learn strategies within the university setting.

*Perceived barriers* to implementations of accommodations.

*Knowledge and training related to concussion* including best practices for return to learn.

Survey questions were developed and grouped according to these constructs. Questions included Likert style questions, multiple choice, and open-ended responses to gather more in depth information on the types of procedures utilized by accessibility offices as well as perceived barriers to implementation. Lastly, the survey was reviewed (NM) to ensure questions possessed specificity towards the functions of accessibility offices, incorporating common language amongst these professionals. The list of survey questions including skip logic can be found in Supplementary material 2.

### 2.3 Data analysis

Responses were grouped according to size of institution colleges (<6,500 students; 6,500–10,000; 10,001–20,000; >20,000) and analyzed using descriptive statistics. A two-tail Welch’s ANOVA with Bonferroni corrections was used to assess mean differences in the data, operating on the assumption of unequal sample sizes and variances (*α* = 0.05). Categorical variables were compared through Chi-Square analyses (*α* = 0.05), with post-hoc analyses of adjusted residuals where appropriate.

Qualitative answers were provided to survey questions #19 and #20. Responses were independently open coded by three authors (CF, NM, KB), followed by an iterative process of discussion and revision until 80% agreement was reached (7, 17). Authors then used a deductive lens to independently form themes, working towards unanimous agreement.

Both quantitative and qualitative responses from participants explored facets of feasibility, therefore, a segregated approach (26) to data analysis occurred by which data types were analyzed in isolation and then converged to form holistic conclusions. Finally, resultant themes were used to create a model to guide RTLU procedures and feasibility.

## 3 Results

### 3.1 Demographics

Eighty-seven sets of data were collected, comprised primarily of public (60%), private (32%) and religious (8%) institutions. Females, males, and non-binary/third gender participants represented 74, 21, and 2% of the sample, respectively. Three percent of participants elected not to disclose their gender. Participants identified as White (79%), Black (7%), Asian (2%), and Other (7%), with 5% not disclosing their race. Forty-eight percent of responses were from small colleges (<6,500 students), followed by 13% (6,500–10,000), 14% (10,001–20,000), and 25% (>20,000). Further demographic details are found in Table 1.

**Table 1:** Demographics of participants by size of institution.

Variable	<6,500 Students	6,500–10,000 Students	10,001–20,000 Students	>20,000 Students	Total
Public	16	9	8	19	52
Private	21	2	2	3	28
Religious	5	0	2	0	7
Gender					
Female	30	9	10	15	64
Male	9	1	2	6	18
Non-binary/third gender	1	0	0	1	2
Prefer not to say	2	1	0	0	3
Ethnicity					
Asian	2	0	0	0	2
Black or AA	3	0	2	1	6
Other	0	1	2	3	6
Prefer not to say	1	1	1	1	4
White	36	9	7	17	69
Age					
18–24	0	0	0	1	1
25–34	9	6	2	5	22
35–44	5	3	4	7	19
45–54	7	0	3	4	14
55–64	15	1	2	4	22
65–74	5	0	0	0	5
Prefer not to say	1	1	1	1	4
Years worked in university-level accessibility					
Mean	10	6	7	10	9
Median	9	5	6	7	8
Min	1	1	1	1	1
Max	25	15	18	26	26
Mode	1	3	3	2	1
Staff that provide accommodations					
Mean	3	4	4	8	4
Median	2	4	4	8	3
Min	1	2	2	1	1
Max	10	6	10	11	11
Mode	1	3	4	11	1

All values are displayed as *n*.

### 3.2 State representation

**Figure 1:**
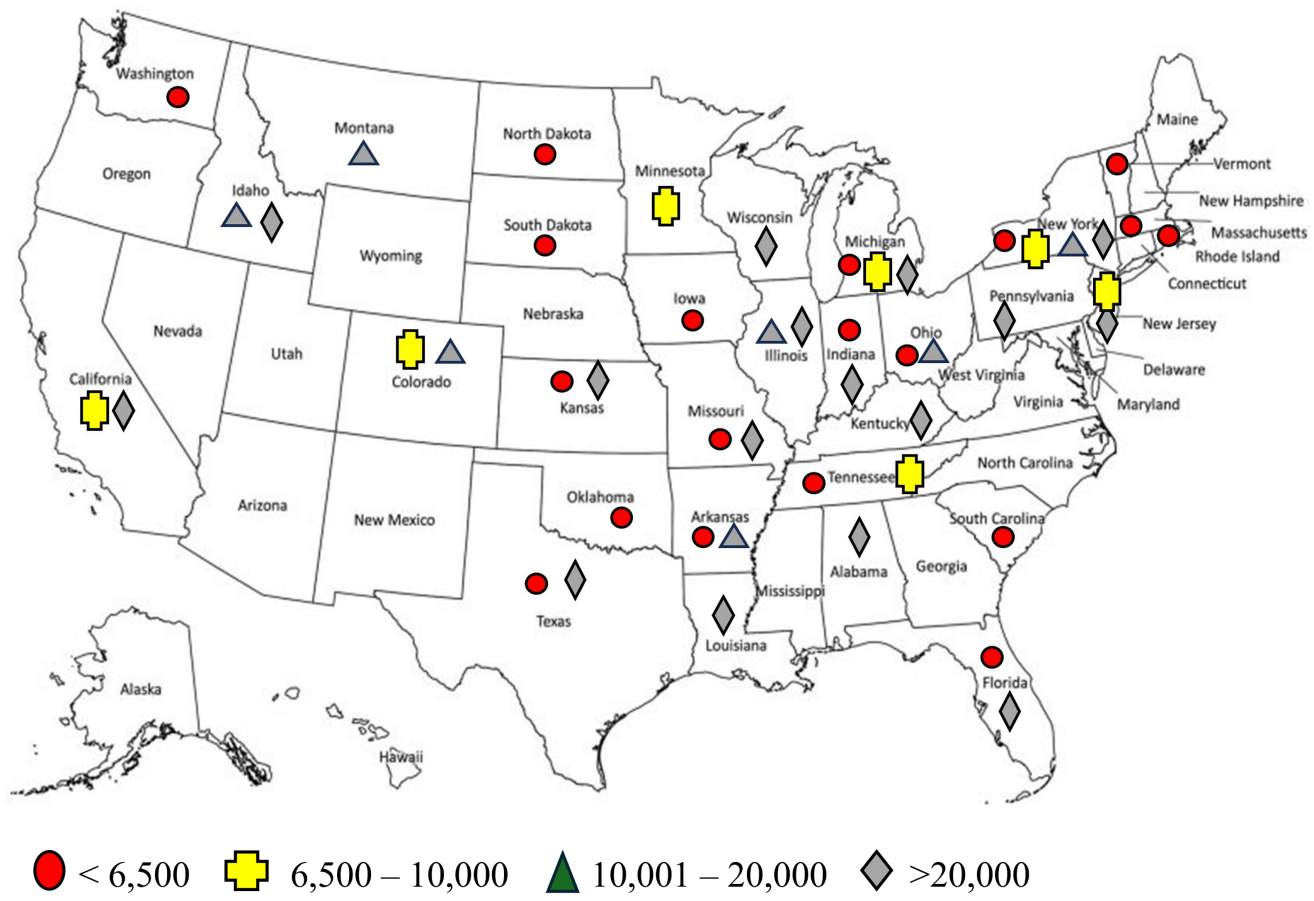
Geographical representation of study participants by size of institution.

Thirty-one United States are represented in the sample. Median and mode number of responses were *n = 2* and *n = 1*, respectively. Table 2 provides the number of responses and category of institution from each state, with Figure 1 displaying the geographical distribution of responses by size of institution.

**Table 2:** Responses by state.

State	Number of responses	Type of institution represented
Alabama	1	Private
Arkansas	3	Private: public
California	4	Public
Colorado	4	Private: public
Florida	3	Public
Idaho	2	Public
Illinois	2	Private: public
Indiana	2	Private: public
Iowa	1	Public
Kansas	2	Public: religious
Kentucky	1	Public
Louisiana	1	Public
Massachusetts	2	Private: public
Michigan	4	Public
Minnesota	1	Public
Missouri	2	Public
Montana	1	Public
New Jersey	3	Public
New York	30	Private: public: religious
North Dakota	2	Religious
Ohio	2	Private: public
Oklahoma	1	Public
Pennsylvania	2	Private: public
Rhode Island	1	Private
South Carolina	1	Private
South Dakota	1	Public
Tennessee	2	Private: religious
Texas	2	Public
Vermont	2	Private: public
Washington	1	Private
Wisconsin	1	Public

### 3.3 Time to implementation of accommodations

Participants responded to the following survey question, “On average, how long does it take to provide ADA and 504 accommodations for a student with a documented temporary disability? (e.g., concussion, broken limb)” (survey question #14). Answers were arranged on a 5-point scale (<1 day, 1–2 days, 3–4 days, 5–6 days, 1 week or more). Seventy percent of all participants selected <1 day or 1–2 days. When grouped by size of institution, the lowest percentage of <1 day or 1–2 days selections were seen by institutions >20,000 students (57%), with the highest percentage seen by institutions <6,500 students (78%) (78, 64, 75, 57%, with increasing size of institution). Chi-Square analysis of time to implementation versus school size was non-significant, *X*^2^ [(12, *N* = 84) = 17.65, *p* = 0.12]. The number of responses from individual states were skewed with NY representing 35% of all responses, thus, a Chi-square analysis was performed to determine whether there was a significant association between the distribution of responses and location (NY, non-NY). Results showed no significant association between variables, *X*^2^ [(4, *N* = 84) = 3.644, *p* = 0.456].

Eighty-nine percent of all participants selected a timeframe ≤4 days. When grouped by size of institution, smaller schools again reported the highest percentage (100, 82, 83, 76%, with increasing size of institution). A significant association between time to implementation (≤ 4 days, > 4 days) and school size was found *X*^2^ [(3, *N* = 84) = 9.650, *p* = 0.022]. Post-hoc analysis using two-tailed pairwise comparisons with Bonferroni corrections yielded significant results (*α* = 0.006), with more participants from schools of <6,500 students reporting timeframes ≤4 days, and fewer participants from these schools reporting timeframes >4 days. Likewise, fewer participants than expected from all other school sizes reported timeframes ≤4 days. Chi-square between location (NY, non-NY) and timeframe (≤4 days, >4 days) revealed no significant association, *X*^2^ [(1, *N* = 84) = 0.675, *p* = 0.411].

### 3.4 Perceived feasibility of implementing accommodations

**Figure 2:**
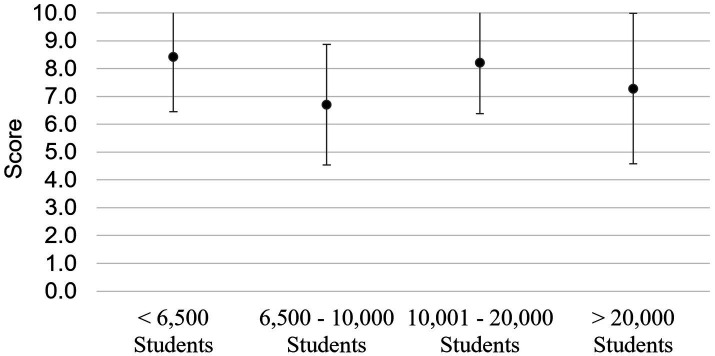
Feasibility of accommodating concussion in <3 days by size of institution. Score ranges from 0 (low feasibility) to 10 (high feasibility). Values are means with standard deviations.

Participants responded to the following survey question, “Concussion research suggests that providing reasonable 504 accommodations to college/university students within days after their concussion (i.e., ≤3) is optimal for ensuring students receive classroom support from faculty. On a scale from 0–10 (0 = not feasible at all; 10 = very feasible), how would you rate this approach at your college/university?” (survey question #27). Thirty-one percent of the total sample believe it is very feasible (10/10 score) to implement accommodations for a concussion in ≤ 3 days. Eighty-one percent scored the feasibility of this task as an 7/10 or greater. Chi-square analysis compared the observed number of scores ≥7/10 versus <7 by size of institution, revealing a significant association, *X*^2^ [(3, *N* = 77) = 11.811, *p* = 0.008]. Post-hoc analysis using two-tailed pairwise comparisons with Bonferroni corrections yielded significant results (*α* = 0.006), indicating scores <7 were fewer than expected while scores ≥7 were more than expected in schools with <6,500 students. A second Chi-square analysis compared feasibility scores by location (NY, non-NY). No significant associations between variables were found, *X*^2^ [(1, *N* = 77) = 0.407, *p* = 0.840]. Finally, mean feasibility scores for each institution size were compared (<6,500 = 8.43; 6,500–10,000 = 6.71; 10,001–20,000 = 8.21; >20,000 = 7.29). Two-tail Welch’s ANOVA with Bonferroni corrections resulted in no significant differences in feasibility scores between institution sizes (*p* = 0.094) (Figure 2). Median feasibility scores for each institution size were (<6,500 = 9; 6,500–10,000 = 7; 10,001–20,000 = 8; >20,000 = 8). Total sample mean and median feasibility were 7.88 and 8, respectively.

### 3.5 Barriers to accommodations

**Figure 3:**
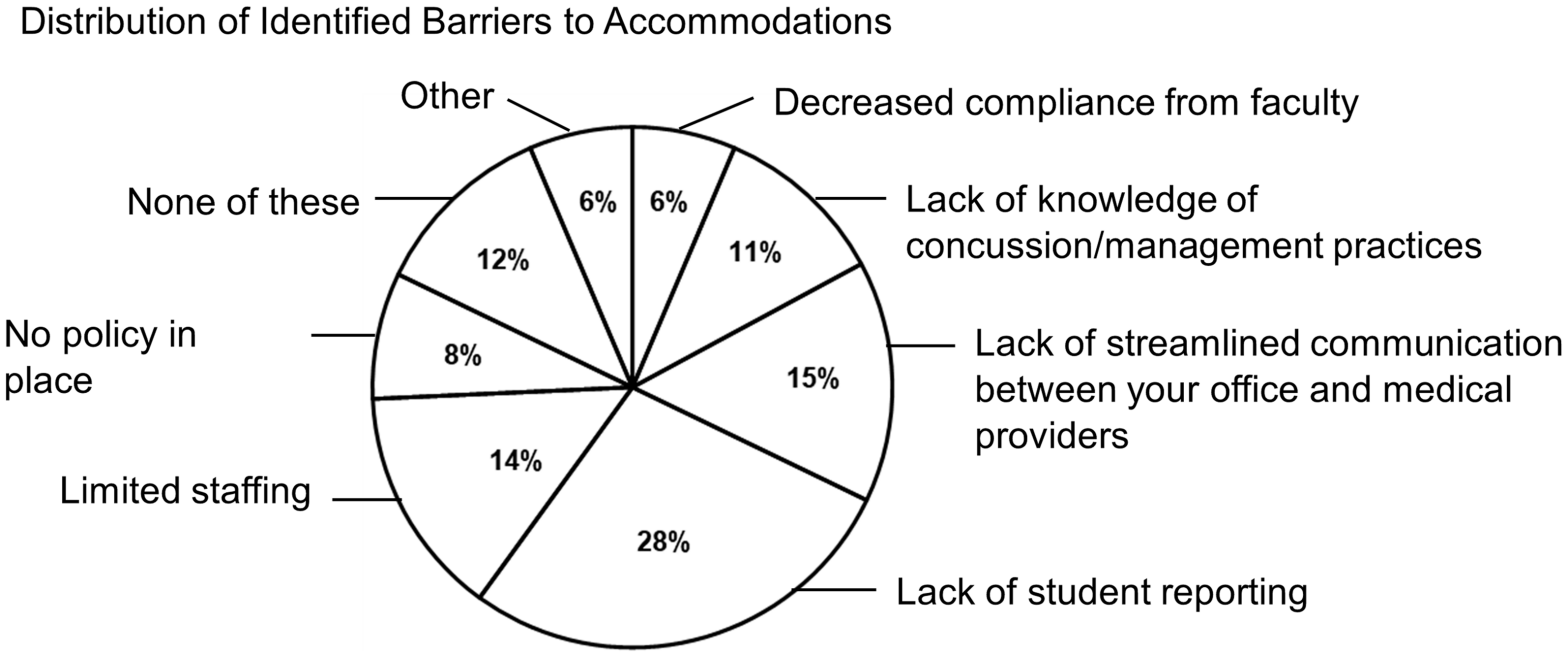
Distribution of identified barriers to accommodations.

Participants responded to the following survey question, “Select all that you believe are current barriers to implementation of accommodations for individuals who have sustained concussions” (survey question #21). Lack of student reporting was the most common barrier selected across the sample (28%), followed by a lack of communication with medical providers (15%), limited staffing (14%), lack of concussion knowledge (11%), no policy in place (8%), and lack of faculty compliance (6%) (Figure 3). Six percent selected “Other,” while 12% identified none of these options as current barriers. Chi-square analysis revealed no significant association between identified barriers and size of institution, *X*^2^ (21, *N* = 140) = 18.23, *p* = 0.635). No association between identified barriers and location were found either (NY, non-NY), *X*^2^ (7, *N* = 140) = 12.30, *p* = 0.091).

Participants also commented on the staffing at their institution, “Please respond to the following statement: I believe my office has enough staffing to provide ADA and 504 accommodations for the students on your campus” (survey question #15). Fifty-seven percent somewhat or strongly disagreed that their institution employs enough staff. Nine percent reported neutrally. When grouped by size, 61% of small institutions somewhat or strongly disagreed, with that number decreasing to 55% at larger schools (61, 55, 50, 55%). Institutions >20,000 students reported the highest percentage of agreement (46%) (32, 27, 25, 46%). There was no correlation between the number of staff and feasibility scores (*r* = 0.015, *p* = 0.899).

### 3.6 Themes

Participants were asked, “Is there anything about your office’s concussion management process that you find to be efficient?” (survey question #20). Four themes emerged from participant responses: speed and responsiveness; standardized procedures; collaboration and communication; specialized care team.

Participants were asked, “Please elaborate on what inspired your scoring above. (scoring 0–10 of accommodating in 3 days or less)” (survey question #28). Six themes emerged: protocol and procedure; documentation; self-reporting; school size and staffing; timely response; faculty flexibility.

### 3.7 Feasibility model for RTLU

**Figure 4:**
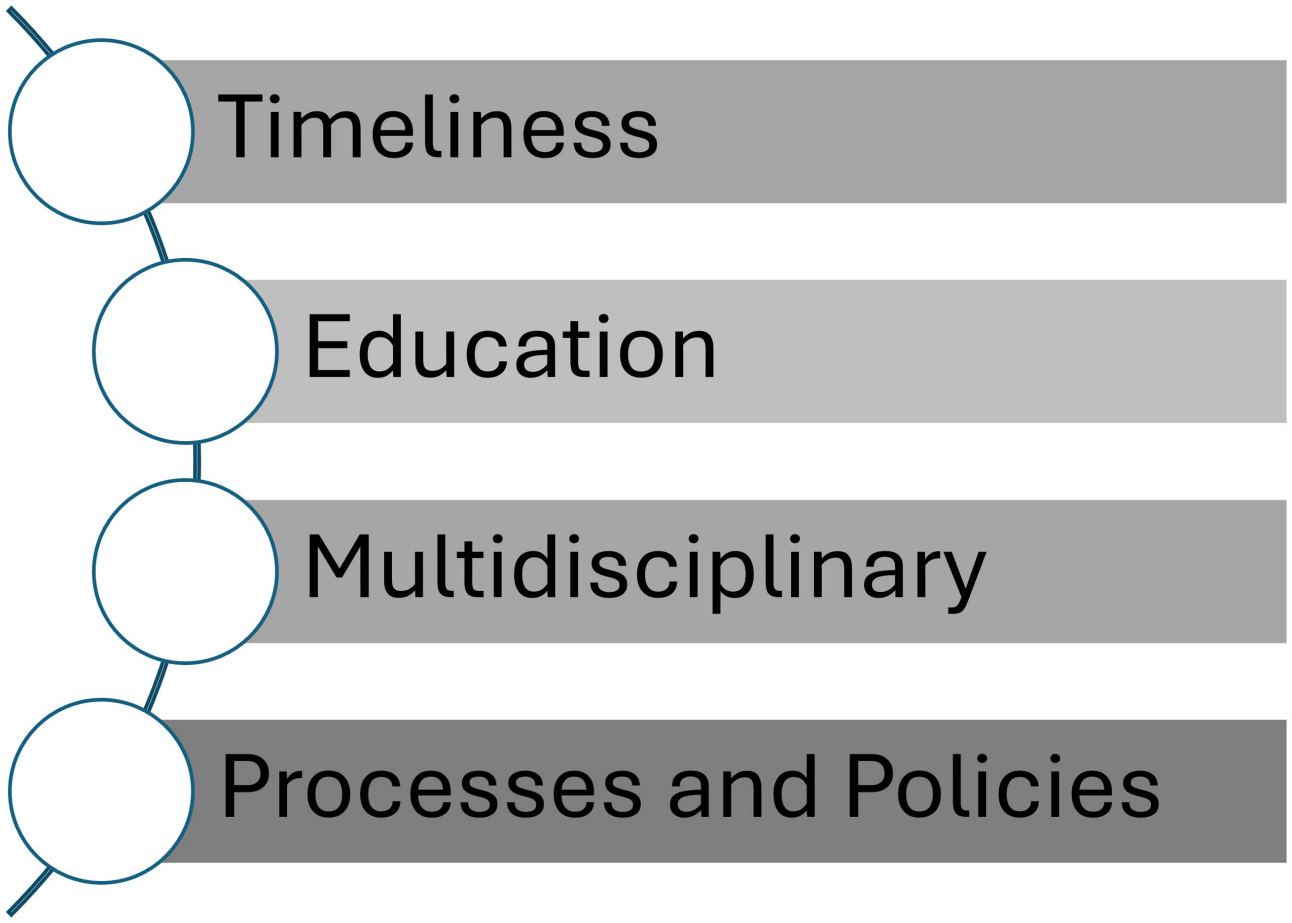
Concussion management at the university level: Framework for Best Practice-TEMP- Model. 1- Timeliness: Accommodations put in place within <3 days 2- Education and training to students, faculty, and staff 3- Multidisciplinary-Collaborative team approach with accessibility staff, faculty, and advisors 4- Streamlined processes and policies: Formalized procedures that are specific to concussion.

Consolidation of themes resulted in the formation of a model for institutions of higher education to utilize when evaluating their RTLU policies and procedures. The model presents four “pillars” that support a feasible RTLU path, including *timeliness*, *education*, *multidisciplinary*, *procedures/policies* (Figure 4. TEMP-Model). *Timeliness* was supported by the themes “speed and responsiveness,” “school size and staffing,” and “timely response.” *Education* was supported by the themes “self-reporting,” and “faculty flexibility.” *Multidisciplinary* was supported by the themes “collaboration and communication,” “specialized care team.” *Procedures/Policies* was supported by the themes “standardized procedures,” “protocol and procedure,” and ‘documentation’.

## 4 Discussion

Results show that accessibility staff perceive accommodations for a concussed student in <3 days as feasible, with 70% of participants currently operating at this pace and nearly all accomplishing this task within 4 days. Limited student reporting, streamlined communication with medical providers, and limited staffing were widely noted as impeding variables.

### 4.1 Perceived contributors to feasibility

Communication and collaboration with medical providers, along with standardized processes were repeatedly noted by participants when describing the efficiencies of their school’s management of temporary disability requests, resulting in the formation of themes (survey question #20). These characteristics provide a framework for what is perceived as an efficient process, though proper documentation and staffing could also adjust the time to accommodations; therefore, rapid accommodations for a temporary disability like concussion appear to benefit from a reproducible process that facilitates communication and the sharing of documents between collaborating professionals, carried out by adequate staffing. Staffing decisions are decided by factors like job duties, campus culture, student population, and academic programs (27), with 133.0 students per disability practitioner as the average national caseload (25). Some have speculated that accessibility offices do not have the resources to manage their caseload and still provide rapid accommodations for concussed students (28, 29). Our findings refute these claims, empirically conveying that this task is quite feasible and already happening. As more students become aware and comfortable with seeking accommodations for concussion, caseloads may grow, though this remains uncertain and highly subject to concussion incidence rates among other variables. Ninety-three percent of respondents confirmed that their office already provides accommodations for concussed students (survey question #18), meaning these students will not represent a new demographic to the existing workflow. Certainly, an appropriate staff-to-student ratio would ease accommodation timelines, though increasing student reporting would have a similar effect. University students must voluntarily seek and pursue accessibility support, helping explain why lack of reporting was noted as the top barrier to rapid accommodations. Low percentages of concussed college students receive accommodations post-injury, (30) leading us to assume that students will often receive medical attention for a concussion but not consult with disability services, particularly if they are unaware of or reluctant to pursue disability support; therefore, direct communication between medical and disability services offers an opportunity to circumvent varying levels of student awareness. Participants agree that a link to medical providers represents an efficient component to rapid concussion accommodations. Mechanisms within the recommended RTLU protocol promote FERPA and HIPAA consent forms as a way to establish a line of communication between medical and accessibility/disability personnel, thereby allowing medical providers to initiate the accommodations process on behalf of the student (22). It is also recommended that medical professionals educate students and promote the value of formal accommodations, especially to students who are apprehensive about reducing academic workloads (14) or identifying as having a temporary disability. Ways of broaching these conversations in-clinic have been introduced, framing accommodations as a tool rather than an inhibition (23). Together, mechanisms like FERPA consent forms that establish collaboration and communication between providers would combat barriers identified by 51% of accessibility staff (i.e., lack of student reporting, 28%; lack of communication with medical providers, 15%; no policy in place, 8%).

### 4.2 Perceived barriers to feasibility

A lack of student reporting was the most frequently identified barrier by participants. Reporting and care-seeking behaviors are multiplicitous in nature, making simple solutions difficult to engineer; therefore, as efforts are made to increase the number of concussed students who receive accommodations, commensurate headway should be made to combat other perceived barriers that could be exacerbated by increasing caseloads, like limited staffing. Only 34% of participants agreed that their institution employs enough staff to support their students, which is curiously juxtapositioned to a high percentage of participants that can facilitate rapid accommodations. No relationship seen between the number of staff and feasibility scores (*r* = 0.015). This suggests that other variables like standardized protocols and communication can drastically attenuate the effects of staffing limitations. Nonetheless, more than half of participants expressed disagreement with having appropriate staffing, echoing a familiar concern (25).

Geographical location was a non-significant variable to several outcome measures. Accessibility offices are governed by civil rights law, thus exercising a wide array of uniformity. It is possible that a larger sample size could produce significant differences between geographical regions, yet the rigid mandates of civil laws encourage us to hypothesize that such differences are reasonably the result of logistical factors, rather than geographical. For example, some participants noted that workflow could be improved with software or an electronic platform through which caseloads are triaged and managed. Programs such as Accommodate by Symplicity® would simplify and streamline day-to-day tasks, but are costly (31).

### 4.3 Feasibility scoring

Feasibility scores were not significantly different across institution sizes. Triangulation with our thematic analysis leads us to conclude that surveyed institutions are benefiting from protocols and collaborations that allow for haste and feasibility, providing clarity to feasibility scores as well as mean and median data. Furthermore, mean and median scores for institution size-based analyses showed tight ranges (1.72 and 2, respectively) suggesting the presence of uniform elements and perspectives across campuses and geographical locations. Redundancy was further seen when analyzing procedures for assisting students (survey question #19), further supporting this conclusion. Last, only 8% of participants identified “no policy” as a barrier, hinting that most are working within a formalized process. These data merge with the perceived contributors outlined previously to affirm standardized protocols, collaboration, and interdisciplinary communication as the uniform variables by which rapid accommodations are facilitated, and triangulate to explain the feasibility scores in Figure 2.

### 4.4 TEMP-model for RTLU feasibility

The TEMP-Model provides a guide for institutions to utilize when evaluating their RTLU policies. Per the model, RTLU procedures should be timely, provide an educational component to users, include multiple disciplines–principally healthcare providers and accessibility staff, and be specific to concussion. Published protocols for higher education are limited, though guidance from Bevilacqua and McPherson (22) lends itself for scrutiny.

The protocol broadly encourages students to seek medical care if they suspect a concussion. However, once a diagnosis is reached, timely implementation of accommodations is emphasized. The protocol follows consensus guidelines to reintegrate academic work no later than 48-h post-injury, thus “timeliness” corresponds to this timeframe. Accommodations are facilitated quickly to meet this demand through a multidisciplinary approach. Students provide permission (i.e., FERPA, HIPAA) for healthcare providers to communicate with the accessibility/disability services office on campus, meaning communication can begin immediately. Additionally, open communication will foster learning opportunities for accessibility staff to discuss and understand why particular accommodations are commonly selected by practitioners to support symptom profiles of concussion. Finally, the protocol is specific to concussion in that it recognizes the categorization of concussion as a temporary disability (23) and correctly utilizes accessibility services as the optimal route for acquiring accommodations. The authoritative position of an accessibility services office further ensures that RTLU procedures are enforceable.

The example protocol possesses clear illustrations of the TEMP-Model, yet it does not explicitly detail educational efforts for students. Concussion education and use of protocols is routine among student athlete populations, though these students represent a fraction of the campus population. To that end, this RTLU protocol could supplement with educational materials, encouraging administrators to disseminate appropriately to students. Positively, brief informational videos are available to quickly convey concussion facts and recommendations tailored for college students (32).

### 4.5 Implications

The findings fill a critical gap in our understanding of what accessibility staff perceive to be a feasible RTLU path. Furthermore, participants’ responses and the TEMP-Model show confidence in the RTLU protocol by Bevilacqua and McPherson. Accessibility staff function as a conduit between medical and academic professionals, but more importantly have identified these connections and collaborations as efficiencies when supporting students with concussion. This confirms recommendations to situate campus accessibility/disability services offices as chief contributors within RTLU (17, 18, 22). On campus, FERPA permission forms are recommended to facilitate instant collaboration between healthcare and accessibility professionals (22), corroborating participants’ description of collaboration as an efficient procedural component, and a barrier when absent. Importantly, accommodations can be feasibly implemented by most staff in <3 days, which agrees with consensus recommendations to re-integrate academics no later than 48 h post-injury (2). This will provide a path towards accommodations like a notetaker (29) and additional time on exam/assignments (33), which have been reported helpful by students. Furthermore, student athletes who receive education on the impacts of concussion on academic performance are significantly more likely to receive accommodations (*p* < 0.05), while a history of concussion drastically increases the odds of using accommodations (OR = 22.5, 95% CI: 2.32, 218.35) (29), suggesting that a growing number of student athletes will seek and utilize accommodations. Overall, these data are promising in that they reveal an existing capacity to augment the RTLU experience for students and underpin the viability of an RTLU protocol.

### 4.6 Limitations

The present study is not without limitations. Although the survey utilized in this study was developed through extensive review of the literature, the survey was not validated. The findings of this study may guide refinement of this survey tool for future studies. Another limitation was that although the sample reflected participants from a variety of geographic regions including 31 states, the sample size was relatively small and may not reflect all accessibility offices throughout the entire country. Similarly, offices with efficient processes may have been more likely to respond while under-resourced offices may have abstained, potentially skewing feasibility scores or reported timelines. Since the survey was disseminated through various methods including public directories and listservs, an objective response rate was not obtained.

## 5 Conclusion

Accessibility staff find rapid accommodations to be a feasible task, revealing an existing capacity to perform RTLU recommendations, assisting students with concussion. The gap remains, however, to educate students about how an Accessibility Office can support their concussion recovery. As procedural links in the RTLU chain are supported with data like these, campuses will be able to confidently spread concussion management education, having efficient mechanisms in place. Therefore, institutions of higher education may find success by adopting recent RTLU guidance (22) while incorporating the campus’ Accessibility Office or like entity as a central component.

## Data Availability

The original contributions presented in the study are included in the article/Supplementary material, further inquiries can be directed to the corresponding author.

## Footnotes

1
https://www.nysdsc.org/
