# Supplementary material for: Rapid accommodations for university students with concussion are feasible: nationwide return-to-learn perspectives of accessibility staff
Source: Front Neurol. 2026 Aug 5;17:1770164. doi: 10.3389/fneur.2026.1770164 (PMC13487392; doi:10.3389/fneur.2026.1770164)
Supplement: Supplementary file 1 [file Table_2.docx]

Survey

Start of Block: Default Question Block

Q1 **Title of research study:** Determining the Role of Accessibility Staff in Concussion Management and College Return-to-Learn. 

 **Investigators and Department:** Zachary Bevilacqua, Ph.D., ATC, Department of Kinesiology, Sports Studies, and Physical Education, SUNY Brockport. Christina Finn, EdD, OTR/L, Department of Occupational Therapy, New York Institute of Technology.

 **Why are we doing this research?** Best practice has yet to be formally establish for managing concussion on college campuses, yet research indicates that accessibility offices could greatly contribute to the academic support of a student recovering from concussion. Therefore, the aims of the proposed study are to: explore accessibility staff perspectives of their current resources (staffing, financial, etc.), and ii) explore staff perspectives on the feasibility of managing academic accommodations associated with concussion management.

 **Why are you being invited to take part in this research study?** We have invited you to take part in this survey study because you are currently employed by an accessibility office on a college or university campus.

**Who can I talk to?** If you have questions, concerns, or complaints, contact Dr. Zachary Bevilacqua, zbevilacqua@brockport.edu. This research has been reviewed and approved by an Institutional Review Board (Protocol #        ). You may talk to them at (585) 395-2472 (Phone) or irb@brockport.edu for any of the following:

- Your questions, concerns, or complaints are not being answered by the research team.
- You cannot reach the research team.
- You want to talk to someone besides the research team.
- You have questions about your rights as a research subject.
- You want to get information or provide input about this research.

**How long does this survey last?** It takes approximately 3-10 minutes depending on your responses and asks simple questions about demographics and your perspectives working in a college/university accessibility office.

 **What are my responsibilities if I take part in this research?** There will be no responsibility regarding participation as this is voluntary, but if you wish to participate in this study, we would appreciate if you answer all survey questions accurately and in-full.

 **What happens if I say no, I do not want to be in this research?** If you decide not to take part in the research there will be no penalty.

 **What happens if I say yes, but I change my mind later?** If you agree to take the survey but change your mind, there will be no penalty.

 **What happens to the information we collect?** Participants may complete the online survey without explicitly identifying themselves. Also, investigators using Qualtrics are advised how to use an anonymous setting such that responses do not include identifying information such as IP address or location information. However, by agreeing to continue, participants acknowledge that NY Tech cannot guarantee anonymity for the survey responses with 100% certainty.

 **By selecting "Yes" below you agree to partake in this survey.**

- Yes
- No

Skip To: End of Survey If Title of research study: Determining the Role of Accessibility Staff in Concussion Management and... = No

Q2 Are you currently working in a college/university Accessibility or Disability Office that provides ADA and 504 accommodations to students?

- Yes
- No

Skip To: End of Survey If Are you currently working in a college/university Accessibility or Disability Office that provide... = No

Q3 Which of these best describes the college or university you work for?

- Private
- Public
- Religious

Q4 Which of these best describes the total enrollment (undergraduate + graduate) of the college or university you work for?

- < 6,500 students
- 6,500 - 10,000 students
- 10,001 - 20,000 students
- > 20,000 students

Q5 Which state is your college or university located?

▼ Alabama ... Wyoming

Q6 What is your sex?

- Male
- Female
- Non-binary / third gender
- Prefer not to say

Q7 What is your ethnicity?

- White
- Black or African American
- American Indian or Alaska Native
- Asian
- Native Hawaiian or Pacific Islander
- Other
- Prefer not to say

Q8 What is your age?

- Under 18
- 18 - 24
- 25 - 34
- 35 - 44
- 45 - 54
- 55 - 64
- 65 - 74
- 75 - 84
- 85 or older
- Prefer not to say

Q9 How many years have you worked in a college/university accessibility office?

▼ 1 ... >40

Q10 Please briefly describe your job duties?

________________________________________________________________

Q11 Including yourself, how many staff members currently work in your office?

▼ 1 ... >10

Q12 Including yourself, how many staff members in your office assist students with reasonable accommodations covered by ADA and 504?

▼ 1 ... >10

Q13 On average, how long does it take to provide ADA and 504 accommodations for a student with a documented disability? (e.g., dyslexia, ADHD).

- < 1 day
- 1-2 days
- 3-4 days
- 5-6 days
- 1 week or more

Q14 On average, how long does it take to provide ADA and 504 accommodations for a student with a documented ***temporary*** disability? (e.g., concussion, broken limb).

- < 1 day
- 1-2 days
- 3-4 days
- 5-6 days
- 1 week or more

Q15 Please respond to the following statement: "I believe my office has enough staffing to provide ADA and 504 accommodations for the students on your campus".

- Strongly disagree
- Somewhat disagree
- Neither agree nor disagree
- Somewhat agree
- Strongly agree

Display This Question:

If Please respond to the following statement: "I believe my office has enough staffing to provide AD... != Strongly agree

Q16 Are there other resources besides staffing that you believe your office requires to support the students on your campus?

________________________________________________________________

| Page Break |  |
| --- | --- |

Q17 How would you define or describe a concussion?

________________________________________________________________

Q18 Does your office provide reasonable accommodations covered by ADA and 504 to students suffering from concussion (i.e., mild traumatic brain injury)?

- Yes
- Unsure
- No

| Page Break |  |
| --- | --- |

Display This Question:

If Does your office provide reasonable accommodations covered by ADA and 504 to students suffering f... = Yes

Q19 Can you describe the procedures utilized by your office when a student sustains a concussion?

________________________________________________________________

Display This Question:

If Does your office provide reasonable accommodations covered by ADA and 504 to students suffering f... = Yes

Q20 Is there anything about your office’s concussion management process that you find to be efficient? Please describe.

________________________________________________________________

Display This Question:

If Does your office provide reasonable accommodations covered by ADA and 504 to students suffering f... = Yes

Q21 Select all that you believe are current barriers to implementation of accommodations for individuals who have sustained concussions.

- Limited staffing
- Lack of knowledge of concussion/management practices
- No policy in place
- Lack of streamlined communication between your office and medical providers
- Decreased compliance from faculty
- Lack of student reporting
- Other __________________________________________________
- None of these

Display This Question:

If Does your office provide reasonable accommodations covered by ADA and 504 to students suffering f... = No

Q22 Please respond to the following statement: "I believe my office should provide reasonable accommodations covered by ADA and 504 to students with a concussion".

- Strongly disagree
- Somewhat disagree
- Neither agree nor disagree
- Somewhat agree
- Strongly agree

Display This Question:

If Does your office provide reasonable accommodations covered by ADA and 504 to students suffering f... = No

Q23 If your office began providing ADA and 504 accommodations for concussion, which of the following would you believe to be barriers to implementation?

- Limited staffing
- Lack of knowledge of concussion/management practices
- No policy in place
- Lack of streamlined communication between your office and medical providers
- Decreased compliance from faculty
- Lack of student reporting
- Other __________________________________________________
- None of these

Display This Question:

If Does your office provide reasonable accommodations covered by ADA and 504 to students suffering f... = Unsure

Q24 Please describe why you are unsure.

________________________________________________________________

Display This Question:

If Does your office provide reasonable accommodations covered by ADA and 504 to students suffering f... = Unsure

Q25 Please respond to the following statement: "I believe my office should provide reasonable accommodations covered by ADA and 504 to students with a concussion".

- Strongly disagree
- Somewhat disagree
- Neither agree nor disagree
- Somewhat agree
- Strongly agree

Display This Question:

If Does your office provide reasonable accommodations covered by ADA and 504 to students suffering f... = Unsure

Q26 If your office began providing ADA and 504 accommodations for concussion, which of the following would you believe to be barriers to implementation?

- Limited staffing
- Lack of knowledge of concussion/management practices
- No policy in place
- Lack of streamlined communication between your office and medical providers
- Decreased compliance from faculty
- Lack of student reporting
- Other __________________________________________________
- None of these

| Page Break |  |
| --- | --- |

Q27 Concussion research suggests that providing reasonable 504 accommodations to college/university students within days after their concussion (i.e., ≤ 3) is optimal for ensuring students receive classroom support from faculty.

 On a scale from 0-10 (0 = not feasible at all; 10 = very feasible), how would you rate this approach at your college/university?

| Feasibility |  |
| --- | --- |

Q28 Please elaborate on what inspired your scoring above.

________________________________________________________________

| Page Break |  |
| --- | --- |

Q29 Have you received education or information on concussion? If yes, please describe what and from where.

________________________________________________________________

Q30 What more would you like to learn regarding concussion and its impact on college students?

________________________________________________________________

| Page Break |  |
| --- | --- |

End of Block: Default Question Block
